# Supplementary material for: HuBIE: The human blood immunome encyclopedia of TCRs and BCRs in bloodstream infections and cancer
Source: Front Immunol. 2026 Jun 18;17:1836543. doi: 10.3389/fimmu.2026.1836543 (PMC13322921; doi:10.3389/fimmu.2026.1836543)
Supplement: Supplementary file 2 [file Table1.pdf]

Supplementary Table 1: Sample categories by participants' conditions

Group	Description	
**Cancers**	The participant's chart contained an ICD-10 code within the prior year indicating any of cancer listed below or participants with a protein electrophoresis result <10 days indicating abnormal gamma bands, suggesting a possible monoclonal gammopathy.	
	breast	lung
	central nervous system	melanoma
	colorectal	ovary
	Hodgkin lymphoma	pancreas
	kidney	prostate
	liver	uterus
	leukemia	monoclonal gammopathy
**Bacterial blood stream infection**	Participants with a recent (within the prior 3 days) blood sample was positive for an infection when cultured in the clinical microbiology lab.	
	Coagulase-negative Staphylococci (CoNS)	*Pseudomonas aeruginosa*
	Enterobacterales	*Staphylococcus aureus*
	Enterococci	Viridans streptococci
**Blood culture controls**	A recent (within the prior 3 days) blood sample was negative for any infection when cultured in the clinical microbiology lab	
**Controls**	Participants who were not in categories above and who did not have any other type of cancer and any type of other bacterial blood stream infection.	
