# Supplementary material for: HuBIE: The human blood immunome encyclopedia of TCRs and BCRs in bloodstream infections and cancer
Source: Front Immunol. 2026 Jun 18;17:1836543. doi: 10.3389/fimmu.2026.1836543 (PMC13322921; doi:10.3389/fimmu.2026.1836543)
Supplement: Supplementary file 3 [file Table2.pdf]

Supplementary Table 2: Additional participants metadata.

Information	Description
**Race**	Self-reported race and/or ethnicity of the participant: White, Hispanic, Black/African-American, Asian or Pacific Islander, Native American, or unknown/not reported/not classified
**Sex**	Gender of the participant according to the medical record as of the blood sample date.
**Age**	Age of participant at time of blood sample; or "90+" whenever the participant was at least 90 years old.
**Antibiotics**	List of medications started before the blood sample date, and either current at the time of the blood sample or discontinued less than 30 days before the blood sample date; number of days given is the number of days before the blood sample date that the medication was started.
**Cancer medication**	List of medications started before the blood sample date, and either current at the time of the blood sample or discontinued less than 30 days before the blood sample date; number of days given is the number of days before the blood sample date that the medication was started.
**Sample dates**	All sample dates were encoded to protect participants' health information.
**Deceased date**	Whether there is a record in the database that the participant has died.
